# Measurement of Switching Performance of Pixelated Silicon Sensor Integrated with Field Effect Transistor

**DOI:** 10.3390/s19245580

**Published:** 2019-12-17

**Authors:** Hyeyoung Lee, Jin-A Jeon, Jinyong Kim, Hyunsu Lee, Moo Hyun Lee, Manwoo Lee, Seungcheol Lee, Hwanbae Park, Sukjune Song

**Affiliations:** 1 Center for Underground Physics (CUP), Institute for Basic Science (IBS), Daejeon 34126, Korea; 2 Department of Physics, Kyungpook National University, Daegu 41566, Korea; 3 Dongnam Inst. of Radiological and Medical Sciences, Jwadong-gil 40, Jangan-eup, Gijang-gun, Busan 40633, Korea

**Keywords:** JFET, pixelated sensor, switching capability

## Abstract

Silicon shows very high detection efficiency for low-energy photons, and the silicon pixel sensor provides high spatial resolution. Pixelated silicon sensors facilitate the direct detection of low-energy X-ray radiation. In this study, we developed junction field effect transistors (JFETs) that can be integrated into a pixelated silicon sensor to effectively handle many signal readout channels due to the pixelated structure without any change in the sensor resolution; this capability of the integrated system arises from the pixelated structure of the sensor. We focused on optimizing the JFET’s switching function, and simulated JFETs with different fabrication parameters. Furthermore, prototype JFET switches were designed and fabricated on the basis of the simulated results. It is important not only to keep the low leakage currents in the JFET but also reduce the current flow as much as possible by providing a high resistance when the JFET switch is off. We determined the optimal fabrication conditions for the effective switching of the JFETs. In this paper, we present the results of the measurement of the switching capability of the fabricated JFETs for various design variables and fabrication conditions.

## 1. Introduction

This paper presents junction field effect transistors (JFETs) that can be used as switches after integration into a pixelated silicon sensor used for the direct detection of low-energy X-ray radiation in protein crystallography. The photon detection efficiency of such a sensor for 10 keV X-rays is about 100% so that the direct detection method without employing a scintillator coupled with a silicon sensor can be used. For these X-rays, the absorption length is 127 μm [1]. Since the thickness of the silicon material should be at least twice the absorption length, a thickness exceeding 254 μm is required [2,3].

**Figure 1:**
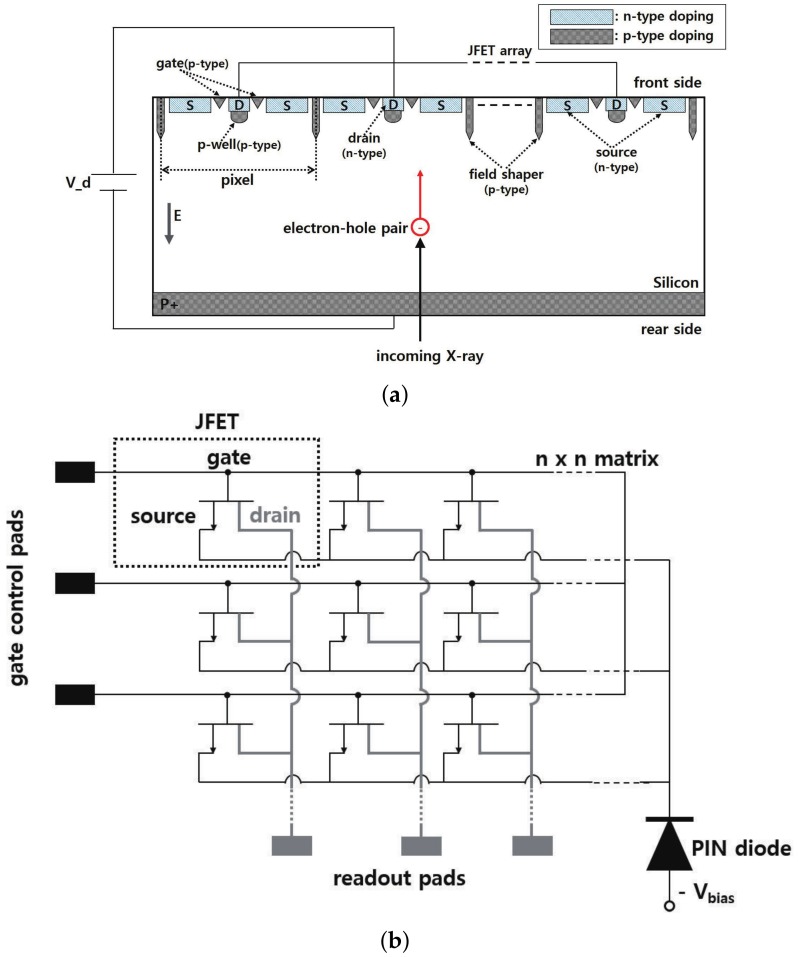
(**a**) Schematic and (**b**) equivalent electronic circuit of a pixelated silicon sensor.

The proposed pixelated silicon sensor, shown in Figure 1a, is capable of directly reading the charges produced after X-rays are converted into electron-hole pairs by the internal photoelectric effect in the silicon material. The number of charges produced is proportional to the number of incident X-ray photons. Apart from facilitating the use of a simple sensor system, the direct detection method prevents the lateral spread of optical photons, which occurs during optical conversion in the indirect conversion detector [4]. The absence of lateral spread reduces the blurring of the signal profile in the direct conversion detector. Owing to the use of JFET technology, the pixelated silicon radiation sensor provides high spatial resolution and shows high detection efficiency for low-energy photons when a silicon material with a thickness in the range of 500–600 μm is used. The JFET is a three terminal, unipolar semiconductor device that can be used as a voltage-controlled switch [5,6].

The fabrication of the rear side of the sensor can affect the front side, where the JFET is located, and therefore, we did not fabricate the rear side, especially since this study focused on the performance of the JFET’s switching capability. The charges produced in the positive-intrinsic-negative (PIN) diode region of the sensor by incident X-rays were attracted to the source terminal of the JFET because of a vertical electric field induced in the diode by the depletion voltage V${}_{dep}$, and they could provide information on the incident point position.

For using the source of the JFET as a capacitor to store charges produced by incident X-rays, we formed an oxide layer, which was a dielectric, at the top of the source. A deep p-well was formed just below the drain, and it covered the drain almost completely. One of the important functions of the deep p-well is to prevent the produced electron-hole pairs from entering the drain terminal directly during charge transfer from the source to the drain terminal [7,8]. Moreover, together with the depletion region of the gate, the depletion region of the p-well plays a major role in the switching operation of the sensor. The gate of the JFET controls the flow of charges from the source to the drain. Specifically, this control is achieved by adjusting the n-channel resistance. Controlling the magnitude of the resistance is not easy, since it involves adjusting the depletion regions of the gate and p-well in the n-channel.

In a pixelated silicon sensor, several hundreds of JFETs can be fabricated on top of a PIN diode of size 1 × 1 cm. One JFET can be defined as one pixel. The JFET size can be varied, and it determines the sensor resolution. We can increase the number of pixels in the X-ray radiation detection sensor by appropriately arranging JFET containing PIN diodes. Figure 1b shows an equivalent electronic circuit of the pixelated silicon sensor [9]. There is an n × n matrix of JFET switches on the front side of the PIN diode. In the matrix, the horizontal lines connected to the gate of each JFET are also electrically connected to the gate control pads, which control the switching of the JFETs. The vertical lines connected to the drain of each JFET are also electrically connected to the bonding pads at the bottom for readout. When an appropriate reverse bias voltage is applied to a gate control pad, all JFET switches (gates) on the line are turned on and charges from their drains are transferred to the readout pad through the vertical read lines. Thus, all the pixels in the row are read out in parallel. After the pixels of a row are read out, the next row is selected by the gate control voltage. By incorporating a JFET in the pixelated silicon sensor, we can easily read up to 100,000 pixels with minimal readout lines.

In this study, for realizing a JFET with high switching capability, we determined the optimal fabrication conditions and design parameters for each terminal (gate, drain, and source) of the JFET. Furthermore, we designed and fabricated JFETs with different sizes (30 × 30 μm, 100 × 100 μm, and 200 × 200 μm). Since it is important to minimize the current flow in the off state of the JFET by providing a high resistance, we simulated, designed, and fabricated different versions of the JFET by varying the doping levels of the device regions, the sizes, and the distances between each of the terminals. The JFETs were tested to ascertain the effects of each variable on the switching and we present the test results in this paper.

## 2. Simulation and Fabrication of JFET

### 2.1. Simulation

**Figure 2:**
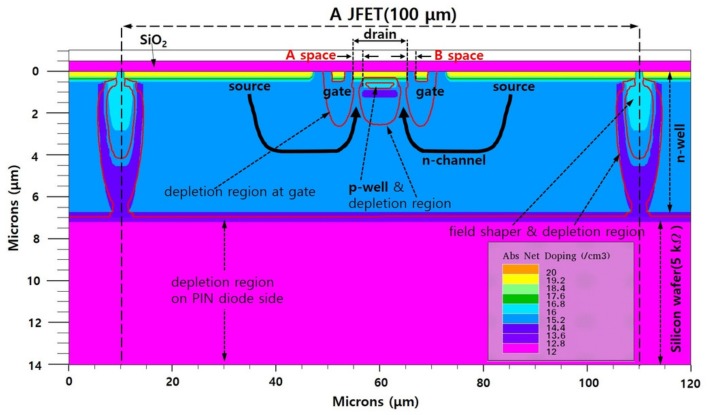
Two-dimensional simulation profile of a junction field effect transistor (JFET) in the pixelated silicon sensor.

The JFET switch structure was simulated using the Silvaco package [10]. Figure 2 shows a cross section of the simulated JFET structure 100 μm in length. The front and rear of the silicon wafer corresponded to the JFET and PIN diode sides, respectively. The JFETs were electrically isolated from each other by deeply formed field shapers [7,11]. The PIN diode side was simulated using an n-type high resistivity ($R_{bulk}$ = 5 kΩ·cm) silicon substrate, while the JFET side was simulated in an n-well formed with a higher doping concentration than the substrate. Since the depletion region cannot easily extend in the n-well ($R_{nwell}$ < $R_{bulk}$), the depletion regions of the gate and p-well in the JFETs were appropriately set.

Each terminal of the JFET was formed with a doping concentration of about $10^{19}$ cm${}^{-3}$ for establishing ohmic contact with a metal pad. As Figure 2 shows, there were depletion regions at the gate, p-well, and field shaper. The depletion region at the gate did not expand when the voltage at the gate terminal was 0 V. When the switch was on, an n-channel was formed between the source and the drain, and it passed through the depletion regions of the p-well and gate.

**Figure 3:**
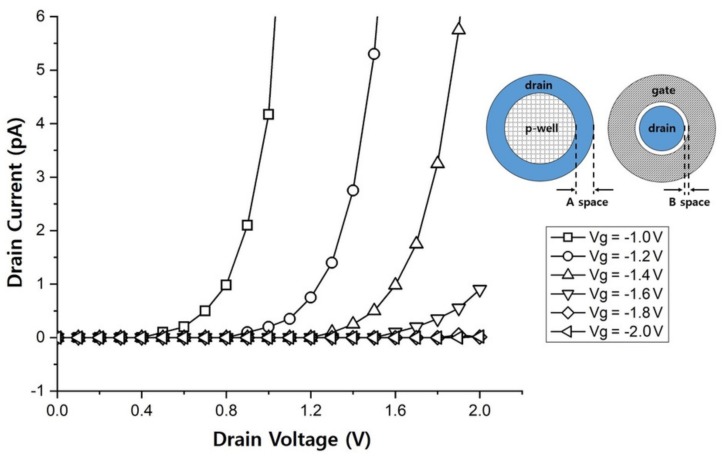
I–V characteristics of a JFET for various gate voltages (V${}_{g}$) in the simulation. The circular drawings show the JFET design parameters: A and B spaces.

Figure 3 depicts an I–V graph with the drain current plotted as a function of the drain voltage for different gate voltages. The graph was obtained from a simulation of the JFET design with an “A space” (difference in the radii of the drain and the deep p-well) of 0.3 μm and a “B space” (distance between the gate and the drain terminals) of 1.0 μm. In the simulation, the drain voltage was varied from 0 to 2 V. The switching functionality was confirmed by applying voltages from –1 to –2 V to the gate terminal. As the reverse bias voltage of the gate was increased, the current flowing through the n-channel drastically decreased. A drain current of about 0.1 pA flowed at the drain voltage of 2 V, indicating a switch-off resistance of about $10^{13}$Ω.

### 2.2. Fabrication

The switching of the JFET depends strongly on the design of the A and B spaces, and it may not occur for specific values of these two parameters. In such a case, a large current may flow even at the gate voltage of –2 V. We first performed a simulation with the A and B spaces as the design parameters and determined the conditions at which the switch functioned well. Subsequently, we designed photomasks that covered a wider range of conditions and compared the results of the simulation with those of the fabricated JFETs.

**Figure 4:**
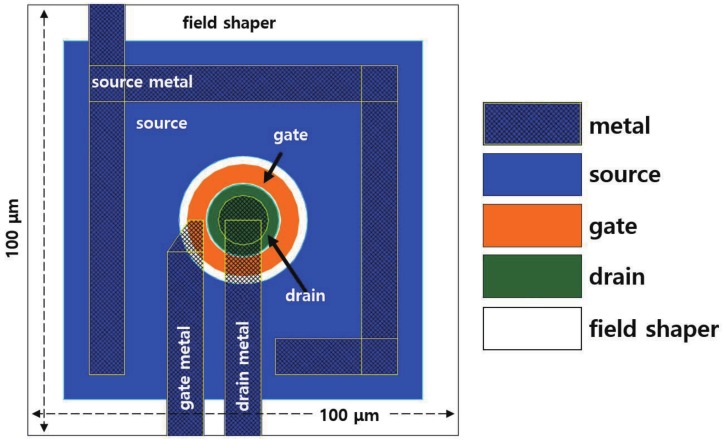
Design of a JFET photomask for the pixelated silicon sensor.

When the size of the matrix of JFET is increased, a two metal layer process is required to form the metal lines for the readout of each terminal [12]. However, it was possible to use only one metal process instead of a two metal layer process by appropriately arranging the metal lines. Apart from reducing the number of photomasks required from 13 to 6, the arrangement decreased the fabrication cost. Figure 4 shows the photomask design for the JFET with a size of 100 × 100 μm. The center of the JFET had a circular drain, and a donut-shaped gate surrounded the drain. The outermost square ring in Figure 4 represents the field shaper. The source was located between the gate and the field shaper. Although not shown in the figure, a circular, deep p-well region was designed with a radius smaller than that of the drain. In order to examine the dependence of the switching performance of a JFET on its size, we designed JFETs with sizes of 200 × 200 μm, 100 × 100 μm, and 30 × 30 μm. We designed the contact and metal pads such that the ground would be connected to the source in order to maintain a potential difference between the source and the drain.

In a fabrication run, we set the doping concentration and depth of each terminal for achieving control over the resistance of the n-channel. We determined the optimal design parameters for the switching of the JFET by splitting the design conditions of the A space and B space in the JFET for three different p-doping concentrations of the p-well under the drain (7.0 $\times 10^{13}$, 7.5 $\times 10^{13}$, and 8.0 $\times 10^{13}$ cm${}^{-3}$).

**Figure 5:**
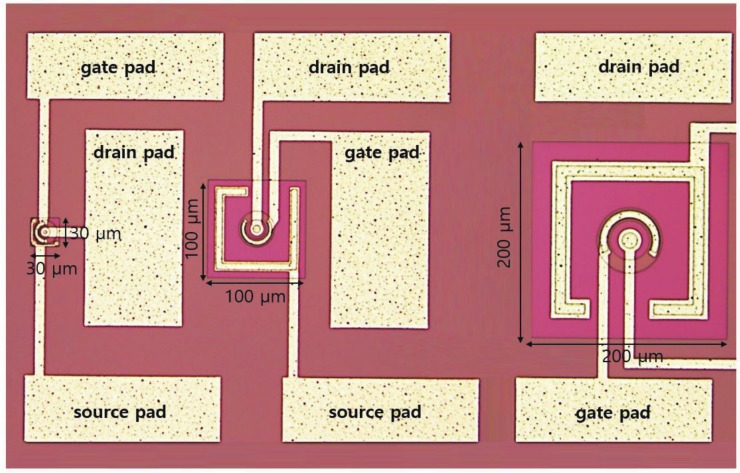
Photograph of junction field effect transistors (JEFTs) of various sizes in the fabricated pixelated silicon sensors.

Figure 5 shows a photograph of a JFETs fabricated on an n-type double-sided polished silicon wafer with a diameter of 6-in, a high resistivity (>5 kΩ·cm), a thickness of 650 μm, and a <100> orientation. The JFET fabrication, performed at the Electronics and Telecommunications Research Institute (ETRI) in Korea, involved a total of 84 fabrication steps of CMOS-specific planar processes, such as oxidation, diffusion, photolithography, ion implantation, and annealing.

## 3. Performance Measurement

When a reverse bias voltage (about –2 V) was applied to the gate, the depletion region at the gate expanded and met the depletion region of the p-well. As the reverse bias voltage was increased, the depletion region at the gate further expanded, increasing the resistance of the n-channel. Consequently, less current flowed in the channel and the JFET was finally turned off.

**Figure 6:**
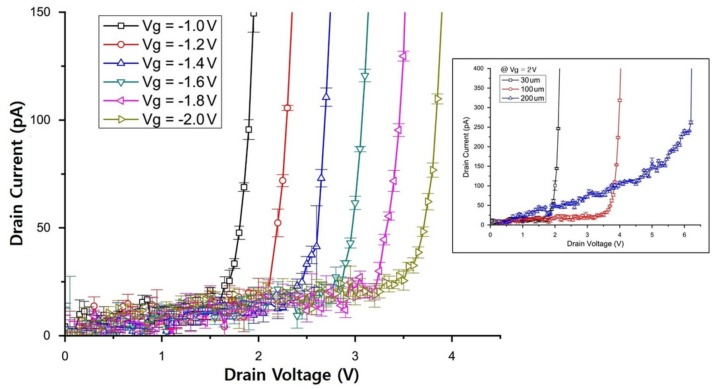
I–V characteristics of the fabricated JFET with an area of 100 × 100 μm for various gate voltages ($V_{g}$). The inset shows the I–V characteristics of JFETs with different sizes but with the same design values of the A and B spaces.

Figure 6 shows an I–V graph of the JFET with a size of 100 × 100 μm and a p+ doping concentration of 7.0 $\times 10^{13}$ cm${}^{-3}$ for the p-well under the drain, for A and B spaces of 0.5 and 1.4 μm, respectively. The drain voltage was increased from 0 to 4 V in steps of 0.1 V and the gate voltage was increased from –1 to –2 V in steps of –0.2 V to check their effects on the switching. It was confirmed that there was hardly any current flow at the gate voltage of –2 V. When the JFET was switched off at the gate voltage of –2 V, the minimum current was greater than the current in the simulation because of the noise level of the current-measurement equipment, which amounted to tens of picoamperes.

We defined the criteria for the normal switching function of a fabricated JFET as follows. When the JFET is switched on, the current should exceed 50 pA at the gate voltage of –1 V, and when the JFET is switched off, the current should less than 50 pA at the gate voltage of –2 V. The switch-off resistance was found to reach about $10^{11}$Ω. Although this value appears to be relatively small, the switching functionality apparently worked well when the gate voltage was between –1 and –2 V. The I–V characteristic graphs for the JFETs with sizes of 30 × 30 μm and 200 × 200 μm with the same design values for the A and B spaces as those for the JFET with a size of 100 × 100 μm are shown in the inset of Figure 6.

**Figure 7:**
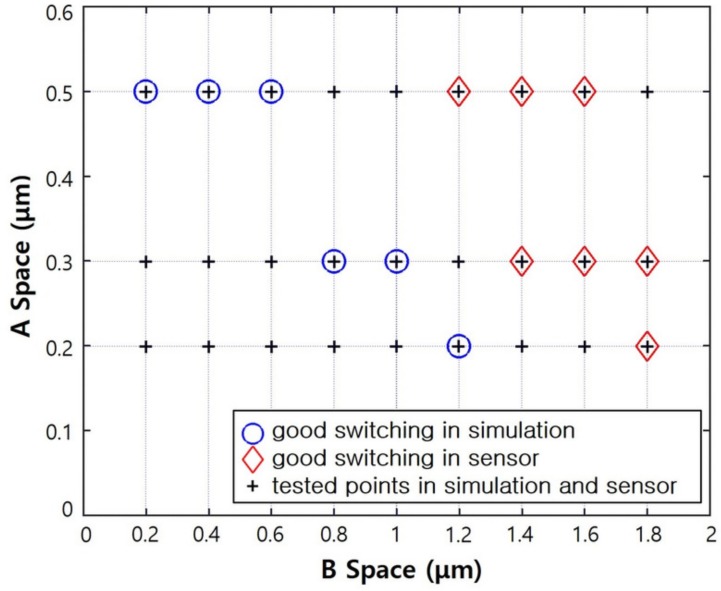
Switching function results of the simulation and for the fabricated JFETs, based on the A and B spaces.

Figure 7 shows the switching capability of the JFET with a size of 100 × 100 μm for different values of the design parameters, A and B spaces. The switching capability of the JFET was determined from the depletion regions at the gate and p-well. The plus marks (+) represent points for which both simulation and fabrication were performed. The circled points (◯) indicate the design parameter values for which the switch functioned well in the simulation, while the diamond marks (◇) indicate the design parameter values for which the switch functioned well in the fabricated JFET sensors. As shown in the figure, a wide range of mask designs were considered: the A space in the JFET could be set to 0.2, 0.3, and 0.5 μm, and the B space could range from 0.2 to 1.8 μm in steps of 0.2 μm. From the simulation results, it is evident that for a given A space, the satisfactory working point of the fabricated switches tended to correspond to a large B space. We understand that this result is due to errors in the actual JFET fabrication. This is because process margins exist in actual fabrication processes. For instance, the margin of a photolithography process equipment (stepper) used in the pattern formation process was typically greater than or equal to 0.2 μm.

Results showing similar trends for the switching performance were obtained under similar design conditions for the JFETs with sizes of 30 × 30 μm and 200 × 200 μm. In the fabrication run, we could identify the design conditions for achieving good switching. Furthermore, we observed that the smaller the area of the pixelated JFET sensor, the less sensitive the results were to the design parameters; namely, the A and B spaces. On the basis of these measurement results, we intend to design more JFET matrices, and the next fabrication run is expected to include a PIN diode process for fabricating the rear side of the pixelated silicon sensor.

## 4. Conclusions

We developed a JFET switch that can be integrated into a pixelated silicon sensor to directly measure low-energy X-rays. It is important to minimize the current flow when JFET is turned off, by providing a high resistance. In this study, the effect of detector design parameters on the switching of the JFET of the sensor was investigated for optimizing the switching performance of the JFET. For this, JFETs were simulated and designed using parameters such as the gate, drain, and p-well doping concentrations and some geometric parameters of the JFET regions as the detector design parameters. We fabricated and tested the JFETs under different design conditions. The dependence of switching on the p-well doping concentration under the drain (7.0 $\times 10^{13}$, 7.5 $\times 10^{13}$, and 8.0 $\times 10^{13}$ cm${}^{-3}$), JFET size (200 × 200 μm, 100 × 100 μm, and 30 × 30 μm), and the distance between switch terminals (up to 0.5 μm for the A space and up to 1.8 μm for the B space) were investigated. At 7.5 $\times 10^{13}$ cm${}^{-3}$ doping concentration level, a change of 10% in the doping concentration had no significant effect on the JFET’s switching performance. The switching efficiency improved with a decrease in the JFET size, and with increases in the A and B space values. The switch-off resistance of the fabricated JFET was found to reach about 10${}^{11}$Ω when the noise level was considered. Although this value appears to be relatively low, the switching functioned well for a gate voltage between –1 and –2 V.
